# Finite Element Methods in Smart Materials and Polymers

**DOI:** 10.3390/polym12061229

**Published:** 2020-05-28

**Authors:** Akif Kaynak, Ali Zolfagharian, Saeid Nahavandi

**Affiliations:** 1 School of Engineering, Deakin University, Geelong, Victoria 3216, Australia; 2 Institute for Intelligent Systems Research and Innovation (IISRI), Deakin University, Geelong 3216, Australia; saeid.nahavandi@deakin.edu.au

**Keywords:** finite element, smart materials, 3D printing, 4D printing, modelling

Functional polymers show unique physical and chemical properties, which can manifest as dynamic responses to external stimuli such as radiation, temperature, chemical reaction, external force, and magnetic and electric fields. Recent advances in the fabrication techniques have enabled different types of polymer systems to be utilized in a wide range of potential applications in smart structures and systems, including structural health monitoring, anti-vibration, and actuators. The progress in these integrated smart structures requires the implementation of finite element modelling using a multiphysics approach in various computational platforms. This special issue presents six scientific report articles.

The Special Issue opens with work from Eindhoven University of Technology in a joint study with Bernal Institute, University of Limerick, Ireland, in which the researchers developed a finite element method (FEM) for representing the microstructural behaviour, particularly the swelling of hydrogel beads [1]. As the design of the cross-linked layers influence the material properties, which, in turn, affect the performance of the hydrogels widely used in pharmaceutical and industrial applications, such as drug delivery or disposable diapers, the authors presented a modelling technique that addressed this limitation for superabsorbent polymers with a partially cross-linked surface layer. The simulations demonstrated that the crack behaviour was influenced by the intrinsic properties of the hydrogel, and provided numerical support for the structural design of the cross-linked hydrogel.

In the second article, Zhang and colleagues from Northeast Agricultural University, China, designed a novel hierarchical metamaterial with a tuneable negative Poisson’s ratio by re-entrant representative star-shaped unit cells [2]. In this work, the in-plane mechanical behaviours of the star-re-entrant hierarchical metamaterial were studied, by FEM, in terms of parameters of cell length, angle of inclination, thickness for star subordinate cell, as well as the amount of subordinate cell along x–y directions. The authors claim that the new hierarchical metamaterial will provide further opportunities to design multifunctional lightweight materials that are promising for various engineering applications in the construction, transportation, aerospace, marine, and manufacturing industries due to the inherent low weight associated with hierarchical systems.

Zolfagharian and Kaynak from the School of Engineering in Deakin University, Australia, worked on the fracture resistance analysis of three-dimensional (3D) printed polymers in collaboration with Khoshravani from the University of Siegen in Germany [3]. The researchers investigated the fracture behaviour of 3D-printed plastic components produced by fused deposition modelling (FDM) and multi-jet fusion (MJF) 3D printing techniques to predict the crack propagation leading to catastrophic failure. U-notched samples manufactured by using nylon and PA12 materials by FDM and MJF printing methods were experimentally analysed. The equivalent material concept (EMC) was used in conjunction with the J-integral failure criterion in ABAQUS software to investigate the failure of the notched samples numerically. Numerical results, supported by experimental analysis, successfully predicted the fracture behaviour of 3D-printed polymer samples. In addition, using the same type of material in the study enabled comparison between the two different printing methods.

In the fourth article, [4], Carleo and colleagues at Queen Mary University of London funded by Jaguar Land Rover investigated the modelling of anti-vibration design in the automotive industry in predicting the dynamic behaviour of the suspension system. In their work, they developed an FEM in ABAQUS software for predicting the viscoelastic behaviour of carbon black-filled rubber as a component of the automotive suspension system. The model used in the study successfully represented the time-dependent phenomenology of filled rubber for use in anti-vibration design considering non-linear elasticity and strain history effects using Maxwell/Prony element and Mullins effect recovery models.

The Special Issue closes with the work of international researchers from the UK, USA, and Egypt in which Atif and colleagues developed a computational fluid dynamics (CFD) model to investigate characteristics of high-speed submicron polyvinylidene fluoride (PVDF) fibres as they are expelled from a blow spinning (SBS) nozzle [5]. The authors used ANSYS Fluent for implementing the CFD model and reported through theoretical and experimental study that a higher air pressure (4 bar) was more suitable to achieve thin fibres of PVDF.
